# Comparison of efficacy of simo decoction and acupuncture or chewing gum alone on postoperative ileus in colorectal cancer resection: a randomized trial

**DOI:** 10.1038/srep37826

**Published:** 2017-01-19

**Authors:** Yang Yang, Hong-Qun Zuo, Zhao Li, Yu-Zhou Qin, Xian-Wei Mo, Ming-Wei Huang, Hao Lai, Liu-Cheng Wu, Jian-Si Chen

**Affiliations:** 1 Department of Gastrointestinal Surgery, Affiliated Tumor Hospital of Guangxi Medical University, Nanning, PR China

## Abstract

To compared the ability of chewing gum or simo decoction (SMD) and acupuncture to reduce incidence of postoperative ileus (POI) after colorectal cancer resection, patients with colorectal cancer undergoing open or laparoscopic resection were randomized to receive SMD and acupuncture (n = 196), chewing gum alone (n = 197) or no intervention (n = 197) starting on postoperative day 1 and continuing for 5 consecutive days. Patients treated with SMD and acupuncture experienced significantly shorter hospital stay, shorter time to first flatus and shorter time to defecation than patients in the other groups (all *P* < 0.05). Incidence of grade I and II complications was also significantly lower in patients treated with SMD and acupuncture. Patients who chewed gum were similar to those who received no intervention in terms of hospital stay, incidence of complications, and time to first bowel motion, flatus, and defecation (all *P* > 0.05). The combination of SMD and acupuncture may reduce the incidence of POI and shorten hospital stay for patients with colorectal cancer after resection. In contrast, chewing gum does not appear to affect recovery of bowel function or hospital stay, though it may benefit patients who undergo open resection. (Clinicaltrials.gov registration number: NCT02813278).

Colorectal cancer resection is one of the most frequent types of abdominal surgery. Though most patients undergoing colorectal resection recover bowel movements within a week, some suffer prolonged intestinal paralysis or postoperative ileus (POI), reducing comfort, increasing morbidity and mortality, and extending hospitalization, all of which increase healthcare costs^1^,^2^,^3^. This highlights the importance of preventing POI^2^,^3^,^4^.

Over the past two decades, many treatments and approaches have been reported for managing POI, including fluid restriction, early enteral nutrition, and nonsteroidal anti-inflammatory drugs^5^,^6^. Randomized controlled trials (RCTs) and systematic reviews have concluded that two traditional Chinese approaches are effective either alone or together for accelerating the recovery of gastrointestinal function after several types of surgery: oral simo decoction (SMD) and acupuncture at the tsusanli acupoint^5^,^7^,^8^,^9^. Chewing gum has emerged as a popular method for reducing the incidence of POI, but its efficacy is controversial. While some systematic reviews and meta-analyses indicate that it can lead to significantly better postoperative bowel function^4^,^10^,^11^,^12^,^13^,^14^,^15^,^16^ and several official guidelines recommend it for preventing POI^17^,^18^,^19^,^20^, three recent RCTs failed to demonstrate an effect of chewing gum on the recovery of bowel function after colorectal resection^21^,^22^,^23^.

Since nearly all previous RCTs of SMD, acupuncture or chewing gum have examined small cohorts, we wished to perform a parallel comparison with a large sample in order to gain more reliable insights into efficacy and safety. In addition, the efficacy of acupuncture alone on the recovery of bowel function was not well defined^7^. And the efficacy of combination of SMD and acupuncture was superior to chewing gum alone^5^. Therefore we conducted this RCT comparing the combination of SMD and acupuncture, chewing gum alone, and no intervention for affecting POI incidence, length of hospital stay, and complications following colorectal resection.

## Methods

The protocol for this trial was designed in accordance with the Declaration of Helsinki and the ethical principles of the International Conference on Harmonization-Good Clinical Practice. The trial protocol was approved by the Medical Ethics Committee of the Affiliated Tumor Hospital of Guangxi Medical University. All study participants provided written informed consent. Data were collected, analyzed and reported according to the Consolidated Standards of Reporting Trials (CONSORT) statement^24^. The trial is retrospectively registered (June 23, 2016) at Clinicaltrials.gov (NCT02813278).

### Patients

Participants were recruited between March 2014 and April 2016 from the two Departments of Gastrointestinal Surgery at the Affiliated Tumor Hospital of Guangxi Medical University (Nanning, China). Patients older than 18 years scheduled for primary colorectal cancer resection, whether laparoscopic or open, were asked to participate. Diagnosis of colorectal cancer was confirmed by histopathological examination of surgical samples. Patients were ineligible if they were younger than 18 years or underwent emergency surgery, had a history of exploratory laparotomy or laparoscopic surgery, had ulcerative colitis or Crohn disease, had a history of abdominal radiation, were pregnant or lactating, were allergic to mint or SMD, required postoperative intensive care for more than 24 h, or were otherwise deemed unsuitable for the study, such as if they had psychological or social conditions that might interfere with their participation.

### Randomization

The trial protocol was explained to all enrolled participants before randomization. After written informed consent was obtained, research staff used TenAlea software (http://nl.tenalea.net) to allocate participants randomly on a 1:1:1 basis to the three arms: SMD combined with acupuncture, chewing gum or no intervention. Randomization was performed the day before colorectal cancer resection. And then, sequentially numbered, opaque sealed envelopes were used. Randomization was stratified by department and, within each department, by resection type (laparoscopy or laparotomy) and disease type (colon or rectal cancer). Patients were informed that the efficacy of SMD, acupuncture, or chewing gum to promote recovery of bowel function after colorectal resection was unknown, and that none of these measures was expected to cause obvious side effects.

### Blinding

Given the different characteristics of SMD, acupuncture, and chewing gum, no blinding was applied to participants or doctors. Nevertheless, the nursing staff and statistician were blinded to treatment allocation throughout data collection and analysis.

### Interventions

All colorectal resections were performed by senior surgeons (length of services ≥ 10 years) using general anesthesia, who consistently applied the same evidence-based, standardized protocols for perioperative management and postoperative care^25^,^26^,^27^. The nasogastric drainage tube was removed on the first postoperative morning. Then the medical team administered the appropriate interventions to each of the three randomized groups. Interventions were recorded in patient records. Nursing staff dispensed SMD and chewing gum to participants every day.

Participants allocated to the SMD + acupuncture group were treated as described^5^. They were asked to take oral SMD decoction (10 mL/dose; Hansen, Yiyang, Hunan, China) three times per day beginning on the first day after colorectal resection. They also received bilateral injections of vitamin B1 (50 mg × 2) at the tsusanli acupoint once per day. This intervention was performed for 5 consecutive days or until flatus. Participants allocated to the chewing gum group were instructed to chew commercially available sugar-free gum (Extra & Reg, Wm. Wrigley Jr., Shanghai, China) three times daily starting on the first postoperative morning. They were instructed to chew the piece of gum for at least 10 min. This intervention was performed for 5 consecutive days or until flatus. Participants in the control group were asked not to undertake any postoperative intervention that might influence recovery of bowel function, including SMD, acupuncture, chewing gum, or adjuvant drugs.

### Acceptability and compliance

Acceptability of SMD + acupuncture and chewing gum was assessed by briefly interviewing participants in these two treatment arms at least 1 full day after resection. They were asked how they felt about the intervention and whether they had any problems or difficulties receiving it. Compliance was assessed by asking participants to record how many SMD doses they took or how long they chewed each piece of gum. Participants in the empty control group were asked at the time of discharge whether they had taken oral SMD or chewed gum during their hospital stay. Participants who discontinued the study or received the incorrect intervention were recorded. All analyses were performed on an intention-to-treat basis.

### Outcome measures

Primary outcomes of this study were time to first bowel motion, time to first flatus and time to defecation, which were obtained from participant questionnaires filled out once daily with assistance from nursing staff who were educated to keep the group allocation secret on days 1–5 after resection. Time to first bowel motion means passage of regular first bowel sounds more than two sounds in every minute first heard on postoperative day^5^,^28^. Secondary endpoints were length of hospital stay, hospital mortality, and postoperative clinical complications such as vomiting, fever, pneumonia, wound infection, and bleeding. Secondary outcomes were assessed by the medical team. Length of hospital stay was calculated as the number of days from the date of colorectal resection to the date of discharge, transfer or death. Criteria for hospital discharge included stable vital signs with no fever, ability to tolerate solid food without nausea or vomiting after defecation, control of postoperative pain, absence of other obvious postoperative complications, and ability to function at home independently or with the home care provided. Those with comorbidity (such as diabetes millitus) will be transfer to another department. Extent of nausea and abdominal pain was reported by participants using a visual analogue scale^29^ once daily on days 1–5 after resection. Postoperative complications were classified and graded according to the Clavien–Dindo classification^30^. Numbers of participants who experienced adverse events were recorded.

### Power calculation and sample size

At the beginning of the study, we did not notice the importance of power calculation for the sample size. Approximately 500 resections for primary colorectal cancer are performed each year in both Departments of Gastrointestinal Surgery at our hospital. To ensure an adequately large sample, we recruited consecutive participants with primary colorectal cancer over two years.

Based on data in the literature, it was assumed that chewing gum could reduce the time to first flatus by 16 h^28^. To achieve a power of 0.8, the sample size for this study was targeted as 42 in each arm. Therefore, the finial sample size (n = 590) was larger enough.

### Statistical analysis

Data for continuous variables were reported using mean (SD) for normally distributed data or median (range) for skewed data. Data for categorical variables were expressed as number (percentage). Intergroup differences were assessed for significance using Student’s *t* test for normally distributed continuous variables or the Mann-Whitney U test for skewed continuous variables. Intergroup differences in categorical data were assessed using the χ^2^ test or Fisher’s exact tests (2-tailed), as appropriate. Length of hospital stay was calculated using Kaplan-Meier analysis and compared between groups using the log-rank test. Data were analyzed using SPSS 19.0 (IBM, USA), with the threshold of significance defined as a two-tailed *P* < 0.05.

Subgroup analysis based on open or laparoscopic resection was performed in order to compare the efficacy of SMD + acupuncture or chewing gum for each type of surgery. In this analysis, patients who underwent laparoscopic surgery that was converted to open surgery were classified as having undergone open resection. Patients who underwent laparoscopically assisted surgery were classified as having undergone laparoscopic surgery.

## Results

### Patient characteristics

**Figure 1:**
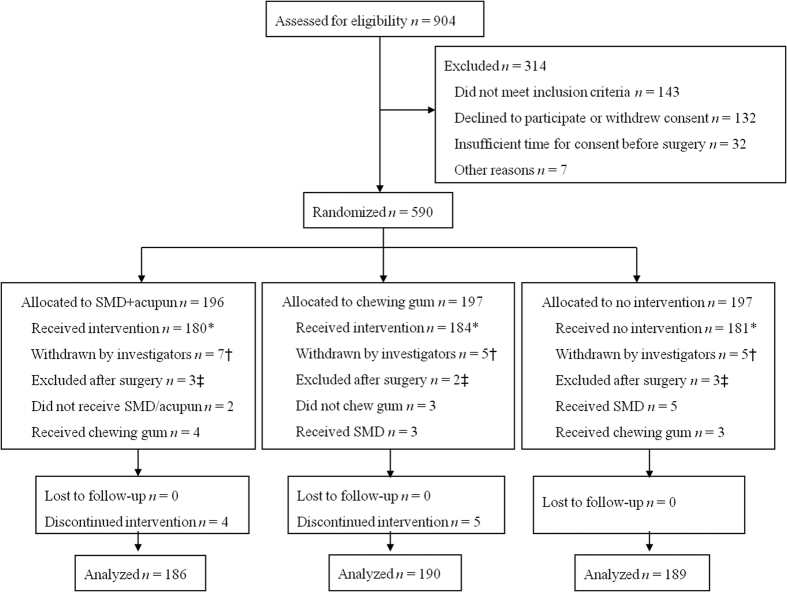
CONSORT diagram for the study. SMD + acupun, simo decoction with acupuncture. *Number of participants who received the allocated intervention. †Withdrawn by investigators before treatment began, because they did not undergo colorectal resection (n = 8), underwent emergency surgery (2), or remained in the intensive care unit for more than 24 h and so could not receive SMD or chewing gum (7). ‡Postoperative pathology revealed benign tumors (n = 8).

From 1 February 2014 to 28 February 2016, 904 patients with primary colorectal cancer were assessed for eligibility. Of these, 143 were excluded because they did not meet the inclusion criteria, 132 declined to participate or withdrew consent, 32 did not have sufficient time to provide consent before surgery, 5 were unwilling to receive acupuncture and 2 were unwilling to receive chewing gum. The remaining 590 participants were randomly assigned to receive SMD + acupuncture (n = 196), chewing gum (n = 197), or no intervention (n = 197). After randomization, 17 patients were withdrawn from the study by investigators before treatment began because they did not undergo colorectal resection, they underwent emergency surgery, or they remained in the intensive care unit longer than 24 h and so could not begin the study intervention in parallel with the other patients. Another 8 patients were excluded after randomization because they were diagnosed with benign tumors based on postoperative pathology. These 25 patients were excluded from the final analysis. In contrast, patients who experienced protocol violations during the study were retained in the final analysis; these violations were failure to receive the planned intervention of SMD + acupuncture or chewing gum (n = 5), administration of the incorrect intervention (n = 15), or patient choice to discontinue the intervention (n = 9). In the end, the final analysis involved 565 patients: 186 in the SMD + acupuncture arm, 190 in the chewing gum arm, and 189 in the no-treatment control arm (Fig. 1). Most patients said that they did not have any problems or difficulties to chew the gum (97.3%) or drink SMD and received acupuncture (97.8%).

Participant characteristics and baseline measures are shown in Table 1. Overall, there were slightly more patients with colon cancer than with rectal cancer, and more than half underwent laparoscopic resection. The three arms were comparable across all demographic and clinical measures. Follow-up on the last participant was 28 May 2016.

### Abdominal pain and nausea

The SMD + acupuncture arm showed significantly lower abdominal pain and nausea scores than the two other arms on day 3 after resection (all *P* < 0.05). The chewing gum and no-intervention arms showed similar scores (Table 2). The three arms showed similar scores on days 4 and 5 (data not shown).

### POI

Participants in the three arms showed similar time to first bowel motion (all *P* > 0.05). In contrast, time to first flatus and time to first defecation were significantly shorter in the SMD + acupuncture arm than in the other two arms (all *P* < 0.05). All three outcomes tended to be shorter in the chewing gum arm than the no-treatment arm, but these differences did not achieve significance (all *P* > 0.05; Table 2).

Within the subgroup of participants who underwent open resection, all three time intervals were significantly shorter in the two intervention arms than in the no-intervention arm (all *P* < 0.05), and all three intervals tended to be shorter in the SMD + acupuncture arm than in the chewing gum arm (all *P* > 0.05; Table 3). Within the subgroup of participants who underwent laparoscopic resection, the three time intervals varied among the three patient arms similarly to how they varied across the entire patient arms (data not shown).

### Length of hospital stay

Hospital stay lasted a mean of 8.9 d (SD 1.9, median 9.0) for patients receiving SMD + acupuncture, 10.5 d (SD 2.5, median 10.4) for patients receiving chewing gum, and 10.9 d (SD 2.4, median 10.5) for no-intervention controls (Table 2). Kaplan-Meier analysis showed that length of stay was significantly shorter for the SMD + acupuncture group than for the other two groups (all *P* < 0.05). Length of hospital stay was similar between the chewing gum and no-intervention groups (*P* = 0.318).

Within the subgroup of participants who underwent open resection, the hospital stay lasted a mean of 9.1 d (SD 2.1, median 9.6) for patients receiving SMD + acupuncture, 10.4 d (SD 2.9, median 10.1) for patients receiving chewing gum, and 11.1 d (SD 3.3, median 11.3) for no-intervention controls (Table 3). Kaplan-Meier analysis showed that length of stay was significantly shorter for either of the two interventions than for no intervention (all *P* < 0.05). Results for the subgroup of participants who underwent laparoscopic resection were similar to those observed across the entire study population (data not shown).

### Complications

Most complications were grade I or II and included wound pain, abdominal distension, fever, and nausea/vomiting. The rate of complications was significantly higher in the no-intervention group than in the other two groups (*P* < 0.001; Table 4). More serious complications requiring pharmacological or other interventions included anastomotic leakage (n = 21), anastomotic bleeding (15), bowel obstruction (18), wound infection (15), pneumonia (8), and death (3) (Table 5). Incidence of serious complications was similar among the three arms, although the rate of bowel obstruction was marginally higher in the no-intervention arm (9 of 189, 4.8%) than in the SMD + acupuncture (3 of 186, 1.6%) or chewing gum arms (6 of 190, 3.2%). All these serious complications were classified as unrelated or likely to be unrelated to the interventions.

## Discussion

A substantial proportion of patients suffers transient impairment of gastrointestinal motility known as POI after abdominal surgery^31^. Since POI increases healthcare costs and resource utilization^32^, investigators have explored various strategies to reduce its incidence, but none is cost-effective^33^. Our results based on a relatively large sample suggest that the combination of SMD and acupuncture significantly enhances bowel function recovery and shortens hospital stay in patients with colorectal cancer after open or laparoscopic resection. Chewing gum may also reduce incidence of POI and affect hospital stay of patients after open resection. However, chewing gum did not significantly enhance bowel function or shorten hospital stay among the entire study population or within the subgroup of those who underwent laparoscopic resection.

The mechanism of POI is complex, characterized mainly by intestinal inflammatory infiltration^34^,^35^,^36^. In traditional Chinese medicine, SMD and acupuncture at the tsusanli acupoint have long been used to reduce risk of POI and manage various functional gastrointestinal disorders^8^,^9^,^37^. The tsusanli acupoint is located on the stomach meridian, and acupuncture there is thought to regulate the intestines. Concurrent administration of vitamin B1 at acupoints is thought to intensify and prolong acupoint stimulation. These considerations may help explain why SMD + acupuncture with concurrent vitamin B1 therapy promoted bowel function to a greater extent than chewing gum or no intervention in our study.

Our results are consistent with randomized trials demonstrating positive effects of SMD and acupuncture on their own or in combination for reducing POI incidence. These benefits have been demonstrated for patients following hepatic resection^5^, gastrectomy^38^, and other surgeries^8^,^9^. We extend the literature by showing, for the first time in colorectal cancer resection, the clinical efficacy of SMD + tsusanli acupoint injection + vitamin B1. At the same time, we did not observe enhancement of bowel function in the entire chewing gum group or in the subgroup of those who underwent laparoscopic resection. This is inconsistent with most randomized trials and meta-analyses on this question^4^,^10^,^11^,^12^,^13^,^14^,^15^. Nevertheless, consistent with this previous work^4^,^10^,^11^,^12^,^13^,^14^,^15^, we did find clinical benefit for chewing gum in the subgroup of those who underwent open resection. Since each treatment arm in the present study was larger than in most previous studies, and since we performed a three-way parallel comparison, our results constitute strong evidence that the combination SMD + acupuncture is likely to provide substantially greater clinical benefit than chewing gum to a larger proportion of patients with colorectal cancer after resection.

The rate of postoperative complications in our study was significantly lower in the two intervention arms, and most complications were grade I or II (Table 4). The rate of serious complications was comparable among the three arms (Table 5), and none of the complications was attributed to the study interventions. Only two of 565 patients in the entire population (0.35%) died within 90 days after surgery, and none of the deaths was attributed to the intervention (chewing gum). These findings are consistent with similar reports showing the safety of SMD, acupuncture and chewing gum after surgery^1^,^2^,^5^,^21^,^22^,^23^. We conclude that SMD, acupuncture, and chewing gum do not significantly affect risk of incidence or type of complications. Moreover, most patients found SMD + acupuncture or chewing gum acceptable and they adhered to the treatment: only 29 of 590 patients (4.9%) received incorrect interventions or discontinued intervention (Fig. 1).

Similarly to our results for the primary endpoints of bowel function recovery, we found that across the entire study population, SMD + acupuncture significantly shortened hospital stay by 2.0 d (8.9 vs. 10.9), while chewing gum reduced it by an insignificant 0.4 d (10.5 vs. 10.9). However, chewing gum did significantly shorten hospital stay in the subgroup of patients who underwent open resection. These results have several possible explanations. One is that SMD + acupuncture stimulates gastrointestinal motility more strongly than chewing gum, thereby accelerating bowel function recovery. Such patients more quickly achieve euphagia without vomiting and begin to ambulate^5^. At the same time, the lower incidence of grade I and II postoperative complications and bowel obstruction in the two intervention arms likely contributed to the shorter hospital stay.

This study has at least three strengths. First, the study population of 590 patients, recruited over two years, is larger than in similar RCTs in the literature. Second, the study population included only patients with colorectal cancer with no history of abdominal surgery, making it more homogeneous than the populations in previous trials that included patients with various types of colorectal disease^2^,^22^,^23^ or with a history of abdominal surgery^2^. Third, in part because of our large sample, we were able to perform subgroup analysis based on open or laparoscopic resection. This allowed us to nuance our finding of no clinical benefit to chewing gum across the entire study population: in fact, chewing gum significantly improved bowel function recovery and shortened hospital stay of patients who underwent open resection. It is possible that this surgery-specific effect reflects the fact that laparoscopic resection, although it usually takes longer than open resection, induces less trauma and stress in the patient. In addition, recovery-enhancing methods are easier to apply after laparoscopic resection because of less trauma^39^,^40^. Thus the clinical benefit of chewing gum may have been too weak to be observed in the entire study population or in the subgroup of those who underwent the laparoscopic procedure. Whatever the explanation, our results suggest, for the first time, that chewing gum may offer clinical benefit only to a subset of patients undergoing surgery. This possibility, which should be verified and extended in future work, is consistent with studies showing that, with the implementation of fast-track surgery in recent decades, chewing gum can be neither clearly recommended nor prohibited as a gastrointestinal stimulant^21^,^22^,^23^.

The present study also has some limitations. One is that length of stay within each arm was calculated over all patients in each arm, regardless of the type of resection that they underwent, which included open, laparoscopic, laparoscopically assisted, and laparoscopic-converted-to-open procedures. This may have confounded the analysis, though the various types of procedures occurred with similar frequencies among the three study arms. A second limitation is lack of blinding for patients and doctors, which was judged impractical because of the nature of the interventions. This limitation is shared with similar trials in the literature^21^,^22^,^23^, and we attempted to compensate for potential bias by blinding the nursing staff to assess primary outcomes and statistician to patient allocation throughout data analysis. A third limitation is that some patients within each arm received opioid analgesia, which may have confounded our analysis^5^. However, the proportions of patients receiving such analgesia were similar among the arms.

Despite these limitations, the present study presents some of the strongest evidence to date that SMD + acupuncture and chewing gum can be safely administered in a postoperative setting to patients with colorectal cancer after resection, and that SMD + acupuncture significantly enhances bowel function recovery and shortens hospital stay, more robustly than chewing gum. Future studies should examine whether SMD + acupuncture or chewing gum can treat POI after it has already developed.

## Additional Information

**How to cite this article**: Yang, Y. *et al*. Comparison of efficacy of simo decoction and acupuncture or chewing gum alone on postoperative ileus in colorectal cancer resection: a randomized trial. *Sci. Rep.* **7**, 37826; doi: 10.1038/srep37826 (2017).

**Publisher's note:** Springer Nature remains neutral with regard to jurisdictional claims in published maps and institutional affiliations.

## Figures and Tables

**Table 1:** Clinicopathological data of patients with colorectal cancer treated by resection (all types) followed by simo decoction + acupuncture, chewing gum or no intervention.

Variable	Simo decoction + acupuncture (n = 186)	Chewing gum (n = 190)	No intervention (n = 189)
Age, yr*	53.7 (15.1)	53.3 (14.9)	54.1 (16.2)
Sex†			
Male	103 (55.4)	106 (55.8)	102 (54.0)
Female	83 (44.6)	84 (44.2)	87 (46.0)
Educational background†			
None or primary school	95 (51.1)	94 (49.5)	98 (51.9)
Secondary school	70 (37.6)	71 (37.4)	72 (38.1)
University degree or above	21 (11.3)	24 (12.6)	19 (10.0)
Body mass index, kg/m^2,^‡	23.4 (18.6–29.3)	23.3 (16.5–29.9)	23.5 (17.1–30.1)
Type 2 diabetes mellitus†	31 (16.7)	32 (16.8)	29 (15.3)
Smoking status†			
Current smoker	30 (16.1)	32 (16.8)	35 (18.5)
Former smoker	31 (16.7)	25 (13.2)	23 (12.2)
Never smoked	125 (67.2)	133 (70.0)	131 (69.3)
ASA fitness grade†			
I	45 (24.2)	43 (22.6)	43 (22.8)
II	120 (64.5)	124 (65.3)	125 (66.1)
III	21 (11.3)	23 (12.1)	21 (11.1)
Indication for resection†			
Colon cancer	103 (55.4)	108 (56.8)	108 (57.1)
Rectal cancer	83 (44.6)	82 (43.2)	81 (42.9)
Type of surgery†			
Laparoscopic	21 (11.3)	19 (10.0)	19 (10.1)
Laparoscopically assisted	118 (63.4)	117 (61.6)	119 (63.0)
Open	40 (21.5)	44 (23.2)	43 (22.8)
Laparoscopic converted to open	7 (3.8)	10 (5.3)	8 (4.2)
Primary procedure†			
Total colectomy	10 (5.4)	10 (5.3)	9 (4.8)
Left-sided colectomy	36 (19.4)	34 (17.9)	32 (16.9)
Right-sided colectomy	48 (25.8)	55 (28.9)	56 (29.6)
Rectal resection	83 (44.6)	82 (43.2)	81 (42.9)
Other§	9 (4.8)	9 (4.8)	11 (5.8)
Surgical time, min‡	141 (60–305)	145 (66—265)	142 (62–271)
Opioid analgesia use†	62 (33.3)	63 (33.2)	57 (30.2)

ASA, American Society of Anesthesiologists.

^*^Values are mean (s.d.).

^†^Values in parentheses are percentages.

^‡^Values are median (range).

^§^Includes partial resection and small bowel resection.

**Table 2:** Outcomes for patients with colorectal cancer treated by resection (all types) followed by simo decoction + acupuncture, chewing gum or no intervention.

Variable	Simo decoction + acupuncture (n = 186)	Chewing gum (n = 190)	No intervention (n = 189)	*P*
Time to first bowel motion, h	17.1 (8.5–41.2)	18.3 (11.0–42.5)	19.1 (10.5–39.4)	0.247* 0.236† 0.265‡
Time to first flatus, h	46.2 (20.5–72.1)	62.3 (21.4–70.5)	64.1 (24.8–71.3)	0.033* 0.021† 0.613‡
Time to first defecation, h	75.2 (29.0–241.6)	119.3 (31.5–211.4)	125.8 (34.2–208.7)	0.042* 0.033† 0.165‡
Length of postoperative hospital stay, d	9.0 (5.3–18.1)	10.4 (6.4–24.1)	10.5 (7.4–21.2)	<0.001*<0.001† 0.113‡
Abdominal pain score on day 3^§^	30 (15–59)	45 (20–79)	49 (23–80)	0.035* 0.027† 0.276‡
Nausea score on day 3^§^	5 (1–50)	9 (2–50)	10 (2–50)	0.039* 0.021† 0.712‡

Values shown are median (range).

^*^Simo decoction + acupuncture vs. chewing gum.

^†^Simo decoction + acupuncture vs. no intervention.

^‡^Chewing gum vs. no intervention.

^§^Visual analogue scale score (in percentage points) on day 3 after surgery; a score of 0 percent means no pain, 100 percent means severe pain or nausea.

**Table 3:** Outcomes for patients with colorectal cancer treated by open resection followed by simo decoction + acupuncture, chewing gum or no intervention.

Variable	Simo decoction + acupuncture (n = 47)	Chewing gum (n = 54)	No intervention (n = 51)	*P*
Time to first bowel motion, h	17.9 (8.9–41.2)	18.1 (11.6–42.5)	21.1 (11.5–39.4)	0.319* 0.226† 0.391‡
Time to first flatus, h	46.9 (21.2–72.1)	50.3 (22.4–70.5)	66.1 (25.3–71.3)	0.074* 0.017† 0.041‡
Time to first defecation, h	75.6 (30.1–241.6)	89.4 (31.5–200.0)	127.2 (35.2–208.7)	0.094* 0.018† 0.037‡
Length of postoperative hospital stay, d	9.6 (6.5–18.1)	10.1 (6.4–24.1)	11.3 (8.4–21.2)	<0.001*<0.001†<0.001‡

Values shown are median (range).

^*^Simo decoction + acupuncture vs. chewing gum.

^†^Simo decoction + acupuncture vs. no intervention.

^‡^Chewing gum vs. no intervention.

**Table 4:** Clavien-Dindo classification of post-resection complications in patients with colorectal cancer treated by resection (all types) followed by simo decoction + acupuncture, chewing gum, or no intervention.

Variable	Simo decoction + acupuncture (n = 186)	Chewing gum (n = 190)	No intervention (n = 189)	*P*
No complications	86 (46.2)	47 (24.7)	9 (4.8)	<0.001*<0.001†<0.001‡
I: deviations from normal postoperative course	66 (35.5)	94 (49.5)	116 (61.4)	0.006*<0.001† 0.020‡
II: complications requiring pharmacological treatment	22 (11.8)	28 (14.7)	44 (23.3)	0.406* 0.004† 0.034‡
IIIa: complications requiring intervention not under general anesthesia	7 (3.8)	10 (5.3)	13 (6.9)	0.484* 0.180† 0.510‡
IIIb: complications requiring intervention under general anesthesia	5 (2.7)	6 (3.2)	8 (4.2)	0.787* 0.414† 0.579‡
IV: life-threatening complications	0 (0)	2 (1.1)	0 (0)	—
V: death	0 (0)	2 (1.1)	0 (0)	—

Values shown are n (%).

^*^Simo decoction + acupuncture vs. chewing gum.

^†^Simo decoction + acupuncture vs. no intervention.

^‡^Chewing gum vs. no intervention.

**Table 5:** Serious postoperative complications in patients with colorectal cancer treated by resection (all types) followed by simo decoction + acupuncture, chewing gum, or no intervention.

Serious complication	Simo decoction + acupuncture (n = 186)	Chewing gum (n = 190)	No intervention (n = 189)	*P*
Pneumonia	1 (0.5)	3 (1.6)	4 (2.1)	0.623* 0.372† 0.724‡
Bowel obstruction	3 (1.6)	6 (3.2)	9 (4.8)	0.503* 0.083† 0.423‡
Wound infection	4 (2.2)	5 (2.6)	6 (3.2)	1.000* 0.751† 0.753‡
Anastomotic leak	6 (3.2)	7 (3.7)	8 (4.2)	0.808* 0.607† 0.784‡
Anastomotic bleeding	4 (2.2)	6 (3.2)	5 (2.6)	0.751* 1.000† 0.766‡
Death	0 (0)	2 (1.1)	0 (0)	—

Values shown are n (%).

^*^Simo decoction + acupuncture vs. chewing gum.

^†^Simo decoction + acupuncture vs. no intervention.

^‡^Chewing gum vs. no intervention.
